# Supplementary material for: Mitotically heritable, RNA polymerase II-independent H3K4 dimethylation stimulates INO1 transcriptional memory
Source: eLife. 2022 May 17;11:e77646. doi: 10.7554/eLife.77646 (PMC9129879; doi:10.7554/eLife.77646)
Supplement: Supplementary file 1. [file elife-77646-supp1.docx]

Supplementary File 1. Yeast strains

| **Strain** | **Genotype** | **Figures** |
| --- | --- | --- |
| CRY1 | *MATa ade2-1 can1-100 his3-11,15 leu2-3,112 trp1-1 ura3-1* | 1B, 2C-E, 2G, 3B, 4E, 7B, F7S1 |
| WLY154 | *MATa ade2-1 can1-100 his3-11,15 leu2-3,112 trp1-1 ura3-1 ino1-mrsmut* | 1B, F6S1 |
| ADY40 | *MATa ade2-1 can1-100 OPI1-GFP-FRB:His5+ leu2-3,112 trp1-1 ura3-1 tor1-1 fpr1∆::NAT RPL13A-2xFKBP12::TRP1* | 1D, 2A, 3C, 5D, 7C |
| BLSY057 | *MATa ade2-1 can1-100 OPI1-GFP-FRB:His5+ leu2-3,112 trp1-1 ura3-1 tor1-1 fpr1::NAT RPL13A-2xFKBP12::TRP1 INO1:mrsmut* | 1D |
| BLSY048 | *MATa ade2-1 can1-100 OPI1-GFP-FRB:His5+ leu2-3,112 trp1-1 ura3-1 tor1-1 fpr1::NAT RPL13A-2xFKBP12::TRP1 Nup100Δ* | 1D, 7C |
| BLSY047 | *MATa ade2-1 can1-100 his3-11,15 leu2-3,112 trp1-1 URA3:pRS306-MCS.1* | 1G, 7D |
| LMY011 | *MATa ade2-1 can1-100 his3-11,15 leu2-3,112 trp1-1 ura3-1 pRS306::URA3* | 1G, 7D |
| BLSY017 | *MATa ade2-1 can1-100 leu2-3,112 trp1-1 his3-11,15 nup100∆::KanMX URA3:pRS306* | 1G, 7D |
| DBY1428 | *MATα ade2-1 can1-100 his3-11,15 leu2-3,112 ura3-1 trp1-1 HTA1-mCherry:HIS5 HIS3:LacIGFP CHO1:p6LacO128* | 1 S2 |
| BLSY086 | *MATa ade2-1 can1-100 OPI1-GFP-FRB:His5+ leu2-3,112 ura3-1 trp1-1 tor1-1 fpr1::NAT RPL13A-2xFKBP12::TRP1 set1∆::KanMX* | 2A |
| DBY1420 | *MATα HTA2-mCherry:HIS5 LacIGFP:LEU2 p6LacO128:INO1* | 3D, 7A, F1S1A |
| DBY1481 | *MATα HTA2-mCherry:HIS5 LacIGFP:LEU2 p6LacO128:INO1 nup100stop-cr* | 3D, F1S1A |
| VSY003 | *MATa ade2-1 can1-100 his3-11,15 leu2-3,112 trp1-1 ura3-1 nup100∆::KanMX* | 2C-D |
| ADY31 | *MATa ade2-1 can1-100 sfl1∆::His5+ leu2-3,112 trp1-1 ura3-1* | 2C, 2E |
| ADY06 | *MATa ade2-1 can1-100 his3-11,15 leu2-3,112 trp1-1 ura3-1 set3∆::KanMX6* | 2E, 2G |
| ICY11 | *MATa ade2-1 can1-100 15 leu2-3,112 trp1-1 ura3-1 swr1∆::His5+* | 2G |
| ADY23 | *MATa ade2-1 can1-100 SWD1-GFP-FRB:His5+ leu2-3,112 trp1-1 ura3-1 tor1-1 fpr1∆::NAT RPL13A-2xFKBP12::TRP1* | 2H |
| BLSY051 | *MATa ade2-1 can1-100 SFL1-GFP-FRB:His5+ leu2-3,112 trp1-1 ura3-1 HMS2:HMS2-AID-9XMYC-HYG* | 3A |
| BLSY052 | *MATa ade2-1 can1-100 SFL1-GFP-FRB:His5+ leu2-3,112 trp1-1 ura3-1 ino1-mrsmut HMS2-AID-9XMYC:HYG* | 3A |
| BLSY042 | *MATa ade2-1 can1-100 his3-11,112 trp1-1 ura3-1 PHO88-mCherry:SpHis5 LEU2:LacI-GFP INO1:p6LacO128 hms2Δ::KanMX* | 3B, 3E, F3S1 |
| BLSY088 | *MATa ade2-1 can1-100 OPI1-GFP-FRB:His5+ leu2-3,112 ura3-1 trp1-1 tor1-1 fpr1::NAT RPL13A-2xFKBP12::TRP1 hms2∆::KanMX* | 3C |
| ADY32 | *MATa ade2-1 can1-100 SFL1-GFP-FRB:His5+ leu2-3,112 trp1-1 ura3-1* | 3D |
| DBY1945 | *MATa Sfl1GFP:HIS5 + hms2∆::KanMX* | 3D |
| JMY049 | *MATa ade2-1 can1-100 his3-11,112 trp1-1 ura3-1 PHO88-mCherry:SpHis5 LEU2:LacI-GFP slf1∆::KanMX INO1:p6LacO128* | 3E |
| ADY70 | *MATa ade2-1 can1-100 his3-11,15 SSN3(Y236G)-GFP:His5+ leu2-3,112 trp1-1 ura3-1* | 4A-D |
| ADY21 | *MATa ade2-1 can1-100 SPT15:His5+ leu2-3,112 trp1-1 ura3-1 tor1-1 fpr1∆::NAT RPL13A-2xFKBP12::TRP1* | 4A, 4D |
| BLSY062 | *MATa ade2-1 can1-100 his3-11,15 SSN3(Y236G)-GFP:His5+ leu2-3,112 trp1-1 URA3:Tir1 Set3-AID-GFP-HYG* | 4F, 4H |
| JMY047 | *MATa ade2-1 can1-100 his3-11,112 trp1-1 ura3-1 PHO88-mCherry:SpHis5 LEU2:LacI-GFP INO1:p6LacO128* | 5A-C, 5E, 7E, F1S1C-E, F3S1, F5S1 |
| BLSY056 | *MATa ade2-1 can1-100 his3-11,112 trp1-1 ura3-1 PHO88-mCherry:SpHis5 LEU2:LacI-GFP INO1:p6LacO128 cdc73∆::KanMX* | 5A, F5S1 |
| BLSY063 | *MATa ade2-1 can1-100 his3-11,112 trp1-1 ura3-1 PHO88-mCherry:SpHis5 LEU2:LacI-GFP INO1:p6LacO128 ctr9∆::KanMX* | 5A, F5S1 |
| BLSY064 | *MATa ade2-1 can1-100 his3-11,112 trp1-1 ura3-1 PHO88-mCherry:SpHis5 LEU2:LacI-GFP INO1:p6LacO128 leo1∆::KanMX* | 5A-C, 7E, F5S1 |
| BLSY065 | *MATa ade2-1 can1-100 his3-11,112 trp1-1 ura3-1 PHO88-mCherry:SpHis5 LEU2:LacI-GFP INO1:p6LacO128 paf1∆::KanMX* | 5A, F5S1 |
| BLSY066 | *MATa ade2-1 can1-100 his3-11,112 trp1-1 ura3-1 PHO88-mCherry:SpHis5 LEU2:LacI-GFP INO1:p6LacO128 rtf1∆::KanMX* | 5A, F5S1 |
| BLSY071 | *MATa ade2-1 can1-100 OPI1-GFP-FRB:His5+ leu2-3,112 trp1-1 tor1-1 fpr1::NAT RPL13A-2xFKBP12::TRP1 Leo1Δ::URA3* | 5D |
| BLSY075 | *MATa ade2-1 can1-100 his3-11,15 leu2-3,112 trp1-1 URA3:pRS306-MRS Leo1Δ::kanMX* | 5E |
| BLSY035 | *MATa ade2-1 can1-100 his3-11,15 leu2-3,112 trp1-1 URA3:Tir1-3XMyc SFL1::Sfl1-AID-GFP-Hyg^r* | 6C |
| DBY1583B | *MATa SFL1::Sfl1-AID-GFP-Hyg^r TetRGFP:LEU2 Tir1:URA3 Pho88mcherry:TRP1 INO1TETO:Nat* | 6B |
| BLSY087 | *MATa trp1-1 ura3-52 prb1-1122 pep4-3 prc1-407 URA3:p6LacO128-MRS SFL1::Sfl1-AID-GFP-Hyg^r LEU2::Tir1 SET3::Set3-AID-FLAG-Nat^r* | 6D-E |
| BLSY034 | *MATa trp1-1 ura3-52 prb1-1122 pep4-3 prc1-407 URA3:p6LacO128-MRS SFL1::Sfl1-AID-GFP-Hyg LEU2::Tir1* | 6D-E |
| BLSY082 | *MATa his3∆1 leu2∆0 met15∆0 ura3∆0 CPS50-TAP:His3* | 6F |
| BLSY080 | *MATa his3∆1 leu2∆0 met15∆0 ura3∆0 SET3-GFP:His3 Cps50-TAP-kanMX* | 6F |
| BLSY081 | *MATa his3∆1 met15∆0 ura3∆0 Set3-GFP:His3 Cps50-TAP-kanMX Leu2:Leo1delete* | 6F |
| BLSY083 | *MATa ade2-1 can1-100 his3-11,15 leu2-3,112 trp1-1 ura3-1 Set3-TAP-kanMX6* | 6F |
| BLSY085 | *MATa ade2-1 can1-100 CPS40-GFP-FRB:His5+ leu2-3,112 trp1-1 ura3-1 Set3-TAP-kanMX6* | 6F |
| BLSY084 | *MATa ade2-1 can1-100 his3-11,15 leu2-3,112 trp1-1 ura3-1 Swd1-TAP-kanMX6* | 6F |
| DBY2016 | *MATa his3∆1 leu2∆1 met15∆0 ura3∆0 NUP100-GFP:His5+ Swd1-TAP::KanMX* | 6F |
| DBY2235 | *MATa/α ade2-1/ ade2-1 can1-100/ can1-100 HIS3:LacI-GFP/ HIS3:LacI-GFP leu2-3,112/LEU2:Tir1-myc trp1-1/TRP1:pPHO88-mCherry ura3-1/URA3:p6LacO128-MRS:KanMX SFL1-AID-myc:Hyg^r^/SFL1-AID-myc:Hyg^r^* | 6S1 |
| JBY461-r2 | *rpb1-1 ade2-1 can1-100 LEU2:UPRE trp1-1 ura3-1 INO1:p6LacO128 HIS3:LacI-GFP SEC63-13myc:KanMX* | F1S1B |
| JMY049 | *ade2-1 can1-100 his3-11,112 trp1-1 ura3-1 PHO88-mCherry:His5+ LEU2:LacI-GFP sfl1∆::KanMX INO1:p6LacO128* | F3S1 |
| BLSY041 | *ade2-1 can1-100 his3-11,112 trp1-1 ura3-1 PHO88-mCherry:SpHis5 LEU2:LacI-GFP INO1:p6LacO128 mga1* | F3S1 |
| BLSY049 | *ade2-1 can1-100 his3-11,112 trp1-1 ura3-1 PHO88-mCherry:SpHis5 LEU2:LacI-GFP INO1:p6LacO128 skn7* | F3S1 |
| DBY1851 | *MATa ade2-1 can1-100 his3-11,112 trp1-1 ura3-1 PHO88-mCherry:SpHis5 LEU2:LacI-GFP INO1:p6LacO128 tatamut* | F1S1C-D, 4E |
