# Supplementary material for: Mitotically heritable, RNA polymerase II-independent H3K4 dimethylation stimulates INO1 transcriptional memory
Source: eLife. 2022 May 17;11:e77646. doi: 10.7554/eLife.77646 (PMC9129879; doi:10.7554/eLife.77646)
Supplement: Supplementary file 2. [file elife-77646-supp2.docx]

Supplementary File 2. Oligonucleotides

| Name | Sequence |
| --- | --- |
| INO1 CDS qPCR REV | TAGTCTTGAACAGTGGGCGTTACAT |
| INO1 CDS qPCR FWD | TAGTTACCGACAAGTGCACGTACAA |
| ACT1 CDS qPCR FWD | TCGTTCCAATTTACGCTGGTT |
| ACT1 CDS qPCR REV | ACCGGCCAATCGATTCTC |
| SNP pRS306 MCS FWD | GTAACGCCAGGGTTTTCCCCGTCACGACGTTGTAAAACG |
| SNP pRS306 MCS REV | CGTTTTACAACGTCGTGACGGGGAAAACCCTGGCGTTAC |
| SNP Comp Check FWD | AAGAAAGCGAAAGGAGCG |
| SNP Comp Check REV | CAGGAAACAGCTATGACCATG |
| INO1Pro TaqMan FWD | TCTTCATCCTTCTTTCCCAGAAT |
| INO1Pro TaqMan Probe | TTCACATGGAGCAGAGAAAGCGCA |
| INO1Pro TaqMan REV | CGAAAGCTCCAATTTATATACGTCTC |
| PRM1cds TaqMan FWD | CGTATTGATATGCTTCGCTGTTG |
| PRM1cds TaqMan probe | CGCCCGTTGCATGGAATGAAATCA |
| PRM1cds TaqMan REV | GCTCAGCATGTAATGGTCTCT |
| Prm1 CDS qPCR FWD | TAACAAGATTTGTCATCCAGCCTGC |
| Prm1 CDS qPCR REV | CCTCCTATACAAAATGGCCAATATG |
| Cdc73-kan PCR insert FWD | AAAAGAATAATAATTTGAGCAAGAAACTGGTGAAAAAATTGACATGGAGGCCCAGAATAC |
| Cdc73-kan PCR insert REV | GACTTCCACATCCTTAAATCTTTTATTTTTGTCAAGTTCGTAATACGACTCACTATAGGG |
| Leo1-kan PCR insert FWD | ATGTCATCTGAAAGCCCACAGGATCAACCACAGAAGGAGCGACATGGAGGCCCAGAATAC |
| Leo1-kan PCR insert REV | TATCATCATTTCCATTGACTTCCTTGACATTATCATCTTGCAGTATAGCGACCAGCATTC |
| Rtf1-kan PCR insert FWD | ATGTCTGATTTAGATGAGGATTTATTAGCCTTGGCTGGTGGACATGGAGGCCCAGAATAC |
| Rtf1-kan PCR insert REV | AATTCACCAACCATGCGCTCCAACCCACCAAGGCGCCTGACAGTATAGCGACCAGCATTC |
| Ctr9-kan PCR insert FWD | ATGACAAACGCAATGAAGGTGGAAGGCTACCCTTCTATGGGACATGGAGGCCCAGAATAC |
| Ctr9-kan PCR insert REV | CATTGTTTTCGTCGTTGTCATCGTTTTTATTTTGTTCCGACAGTATAGCGACCAGCATTC |
| Paf1-kan PCR insert FWD | GTACAATAGAACAGTGCTCATAATAGTATAAAGGGTCACAGACATGGAGGCCCAGAATAC |
| Paf1-kan PCR insert REV | ACAGCATCTGAAGTTTCGGGCGTATCAGCTGCACGGTTTTTAATACGACTCACTATAGGG |
| kanMX insert check REV | TCTGGCGCATCGGGCTTCCCA |
| HMS2 CRISPR FWD | GAGCAAATATAACTTCCAGAGTTTT |
| HMS2 CRISPR REV | TCTGGAAGTTATATTTGCTCGATCA |
| Repair HMS2 CRISPR | GTTAAGCAATTATAACTTCCAGAAGGCGGGCCGCCCGGACTGCGTGGAGTT |
| Ssn3 PntMut Check FWD | GCACAACAAGCATTTAAC |
| Ssn3 PntMut Check REV | TTGTAGCAGATCATGCTC |
| URA3:MRS qPCR FWD | GCCGCGTTGCTGGCGTTTTTCC |
| URA3:MRS qPCR REV | GCCCGCATAGTCAGGAACATCG |
| Hms2 KnockOut FWD | GCAGTCGCCAAGTGTGTCTCTTTCGTGTAGCGACGCACGTCAGACCGGATCCCCGGGTTAATTAA |
| Hms2 KnockOut REV | CGTATATAGTGGTATGCTAGGTCGTGAATGACCCTCAGAGGCAGGGAATTCGAGCTCGTTTAAAC |
| Hms2 KnockOut check | CTAATGGTTGCACAGACGGTAGGCC |
| CPS50AID FWD | ACAATCCCTACAGATTATACGAGGATAATTAAGATGCAGTCATCAcgtacgctgcaggtcgac |
| CPS50AID REV | TCAGTGTCAGTTGCCTATTTAAACTTTCTCCTTTTGAAGAGAAACggccgattcattaatgc |
| FWD MGA1 CRISPR | CTGGTCTCCGACTGATAACAGTTTT |
| REV MGA1 CRISPR | TGTTATCAGTCGGAGACCAGGATCA |
| Repair MGA1 CRISPR | AGTAAATAAATGGATATACTGGTCTTAAACACGGTATTTTTTTTGA |
| FWD SKN7 CRISPR | GGAATGGACTTGTTGCAGAGGTTTT |
| REV SKN7 CRISPR | CTCTGCAACAAGTCCATTCCGATCA |
| Repair SKN7 CRISPR | AGAACAGTTCGACTGCAGACCTTTTAGGATAATCGACTGATGAACACGATGCAGCCAAAC |
| INO1pro tata FWD | GCCGATGTGCCCTTGATGGACAAC |
| INO1pro tata REV | CAAGTCAAGTTTGAACACGTAGTCTTGAACAGTGGGCGTTACATCGAAGCGGGCCACTAGCaGTtTTaGTtACaACAGC |
| FWD SET1-kan | ACGCGTCTTCTGGTTCATACAGACAACCCCCTTGCCTCGTCCCCG |
| REV SET1-kan | AATGGCCGTTTGAATACTGATAGTGACCAGAACTGGATGGCGGCG |
